# Antenatal diagnosis of placenta accreta spectrum after in vitro fertilization-embryo transfer: a systematic review and meta-analysis

**DOI:** 10.1038/s41598-021-88551-7

**Published:** 2021-04-28

**Authors:** Shinya Matsuzaki, Yoshikazu Nagase, Tsuyoshi Takiuchi, Aiko Kakigano, Kazuya Mimura, Misooja Lee, Satoko Matsuzaki, Yutaka Ueda, Takuji Tomimatsu, Masayuki Endo, Tadashi Kimura

**Affiliations:** 1 Department of Obstetrics and Gynecology, Osaka University Graduate School of Medicine, 2-2 Yamadaoka, Suita, Osaka 565-0871 Japan; 2 Department of Gynecology, Osaka International Cancer Institute, Osaka, Japan; 3 Department of Obstetrics and Gynecology, National Cerebral and Cardiovascular Center, Osaka, Japan; 4 Department of Health Science, Osaka University Graduate School of Medicine, Osaka, Japan

**Keywords:** Epidemiology, Outcomes research

## Abstract

Increasing evidence suggests a relationship between in vitro fertilization-embryo transfer (IVF-ET) and placenta accreta spectrum (PAS). Some studies have reported a lower rate of antenatal diagnosis of PAS after IVF-ET compared to PAS with spontaneous conception. This study aimed to review the diagnostic accuracy of PAS after IVF-ET and to explore the relationship between IVF-ET pregnancy and PAS. According to the PRISMA guidelines, a comprehensive systematic review of the literature was conducted through August 31, 2020 to determine the effects of IVF-ET on PAS. In addition, a meta-analysis was conducted to explore the relationship between IVF-ET pregnancy and PAS. Twelve original studies (2011–2020) met the inclusion criteria. Among these, 190,139 IVF-ET pregnancies and 248,534 spontaneous conceptions met the inclusion criteria. In the comparator analysis between PAS after IVF-ET and PAS with spontaneous conception (*n* = 2), the antenatal diagnosis of PAS after IVF-ET was significantly lower than that of PAS with spontaneous conception (22.2% *versus* 94.7%, *P* < 0.01; < 12.9% *versus* 46.9%, *P* < 0.01). The risk of PAS was significantly higher in women who conceived with IVF-ET than in those with spontaneous conception (odds ratio [OR]: 5.03, 95% confidence interval [CI]: 3.34–7.56, *P* < 0.01). In the sensitivity analysis accounting for the type of IVF-ET, frozen ET was associated with an increased risk of PAS (OR: 4.60, 95%CI: 3.42–6.18, *P* < 0.01) compared to fresh ET. Notably, frozen ET with hormone replacement cycle was significantly associated with the prevalence of PAS compared to frozen ET with normal ovulatory cycle (OR: 5.76, 95%CI 3.12–10.64, *P* < 0.01). IVF-ET is associated with PAS, and PAS after IVF-ET was associated with a lower rate of antenatal diagnosis. Therefore, clinicians can pay more attention to the presence of PAS during antenatal evaluation in women with IVF-ET, especially in frozen ET with hormone replacement cycle.

## Introduction

Placenta accreta spectrum (PAS) is associated with an increased maternal morbidity and mortality due to massive hemorrhage during cesarean delivery^1–3^. The mean blood loss during cesarean delivery for PAS is approximately 3000 mL, and the hysterectomy rate is around 40%^4,5^. The main risk factor of PAS is placenta previa, with an approximate odds ratio (OR) of 50–100^6–8^. Several risk factors have been linked to PAS, including a history of cesarean deliveries (OR: 5–9), uterine surgery (OR: 2–3), multiparity (OR: 3), and advanced maternal age (OR: 2.1)^7,9^. Recently, mounting evidence have suggested that in vitro fertilization-embryo transfer (IVF-ET) is associated with PAS, and the OR of PAS is roughly between 3 and 14^10–13^.

IVF-ET currently accounts for 1–4% of live births in the United States and Europe, and the rate has been rapidly increasing as experience with the procedure accumulates and success rates improve^14^. Similarly, the number of PAS after IVF-ET may be increasing^15^. Therefore, clinicians should pay attention to patients with PAS after IVF-ET; despite this, little is known about the characteristics of PAS after IVF-ET^15^. Moreover, to the best of our knowledge, no systematic reviews focusing on PAS after IVF-ET have been performed. A recent study has shown that the diagnostic accuracy of PAS by magnetic resonance imaging (MRI) is lower in PAS after IVF-ET than in PAS with spontaneous conception^15^. Therefore, there is a possibility that PAS after IVF-ET has different characteristics, compared to PAS with spontaneous conception.

Several studies have reported that preoperative assessment of PAS disorders and multidisciplinary surgical approaches are needed to decrease the surgical morbidity of patients with PAS^1,16–18^. Moreover, it has been widely known that the antenatal diagnosis of PAS helps to reduce hemorrhagic morbidity and improves the prognosis; this may be attributed to the comprehensive multidisciplinary care received by patients, which includes a planned cesarean hysterectomy^19–22^.

This study focused on assessing whether the diagnostic accuracy for PAS after IVF-ET is lower than that for PAS with spontaneous conception. Furthermore, a meta-analysis was conducted to explore the relationship between IVF-ET pregnancy and PAS.

## Materials and methods

### Approach for a systematic literature review

A systematic review was performed to review the diagnostic accuracy of PAS after IVF-ET comparing with PAS with spontaneous conception. This study also aimed to examine the effect of IVF-ET on the prevalence of PAS.

### Eligibility criteria, information sources, search strategy

We conducted a systematic search of articles published through August 31, 2020 using PubMed, Scopus, and Cochrane Central Register of Controlled Trials as performed in our previous study^2,23–26^. We reviewed articles according to the Preferred Reporting Items for Systematic Reviews and Meta-Analyses guidelines^27^. Studies were identified by screening the titles, abstracts, and full texts of relevant articles, as previously described. All abstracts were screened by Sh.M. and Y.N.

The following terms were applied in PubMed, Scopus, and the Cochrane database to identify studies which examined the association between IVF-ET and PAS (Supplemental Table S1; MeSH terms were used in PubMed and Cochrane database search): fertilization in vitro [MeSH] OR Assisted Reproductive Techniques [MeSH Terms] OR Embryo Transfer [MeSH Terms] OR Intracytoplasmic Sperm Injection [MeSH Terms] OR "in vitro fertilization" OR “Cryopreserved” OR “Oocyte donation” OR "fresh cycle" OR "frozen cycle" were used to identify the studies regarding IVF-ET.

Studies investigating the effect of IVF-ET on PAS were then identified from this list using the following keywords: Placenta accreta [MeSH Terms] OR "Morbidly adherent placenta" OR "Morbid adherent placenta" OR "Placenta Accreta Spectrum" OR “placenta increta” OR “placenta percreta” OR "adherence of placenta" OR "adherence of the placenta" OR "adherent placenta."

### Study selection

The inclusion criteria based on Patient/Population, Intervention, Comparator, Outcome, Study (PICOS) design are shown in Table 1^28^. Studies were included if they met the following criteria: (1) the effect of IVF-ET on the risk of PAS or antenatal diagnostic accuracy for PAS or maternal outcome for PAS during cesarean delivery was examined; and (2) comparative study was performed between an experimental group and a control group (e.g., IVF-ET *versus* spontaneous conception, frozen ET *versus* fresh ET, etc.).

**Table 1:** PICOS criteria for inclusion of systematic review.

Population	Pregnant women conceived with spontaneous conception and IVF-ET
Intervention	IVF-ET
Comparison	IVF-ET *versus* spontaneous conception Fresh ET *versus* spontaneous conception Frozen ET *versus *spontaneous conception Fresh ET *versus* frozen ET FET with hormone replacement cycle *versus* FET with normal ovulatory cycle
Outcome	Diagnostic accuracy of PAS after IVF-ET compared to PAS with spontaneous conception The relationship between IVF-ET pregnancy and PAS Maternal outcome of PAS after IVF-ET
Study design	Retrospective or prospective cohort studies, case–control study, and randomized controlled trials

*PICOS* patient/population, intervention, comparator, outcome, study, *PAS* placenta accreta spectrum, *IVF-ET* in vitro fertilization-embryo transfer, *ET* embryo transfer, *FET* frozen ET.

The exclusion criteria were as follows: (1) insufficient information about the outcome of interest; (2) studies lacking control arm; (3) article not written in the English language; and (4) conference abstracts, case reports, case series, reviews, systematic review, and meta-analysis.

### Data extraction

Data were extracted by the author (Sh.M. and Y.N.) and the following variables were recorded: PAS type, year of study, first author’s name, study location, number of included cases, the definition of PAS, and outcomes of interest (the risk of PAS, maternal outcome, diagnostic accuracy for PAS).

### Outcome measures analysis and assessment of risk of bias

The primary objective of the study was to review the diagnostic accuracy of PAS after IVF-ET. Two secondary objectives were also examined. First, the relationship between IVF-ET pregnancy and PAS was examined, comparing women who conceived with IVF-ET *versus* those with spontaneous conception. In the sensitivity analysis, the risk of PAS was examined according to the approach of IVF-ET (fresh ET *versus* spontaneous conception, frozen ET *versus* spontaneous conception, fresh ET *versus* frozen ET, frozen ET with hormone replacement cycle *versus* frozen ET with normal ovulatory cycle). Second, maternal outcome of PAS after IVF-ET was examined.

Risk of bias assessment was performed using the Risk Of Bias In Non-randomized Studies-of Interventions tool (ROBINS-I)^29–31^.

### Meta-analysis plan

From the eligible study data, the risk of PAS estimates for the experimental and control groups was computed by using the 95% confidence intervals (CIs) of the reported values to estimate the odds ratios for the risk of PAS. Since most studies reported the odds ratio for PAS, the study which showed the risk ratio without showing crude data was excluded from the analysis. Heterogeneity across the studies was examined using I^2^ statistics, which measures the percentage of total variation across studies. The meta-analysis and the production of all graphics were performed using RevMan ver. 5.4.1 software (Cochrane Collaboration, Copenhagen, Denmark). For consistency, data from all outcomes (continuous and bivariate) were entered into RevMan ver. 5.4.1 in such a way that negative effect sizes or relative risks of < 1 favored active intervention.

### Statistical analysis

Differences in baseline demographics between the two groups were assessed with Fisher exact test, or chi-square as appropriate. All statistical analyses were based on two-sided hypotheses, and a *P*-value of less than 0.05 was considered statistically significant. Statistical Package for Social Sciences (IBM SPSS, version 27.0, Armonk, NY) was used for the analysis.

### Ethical committee exemption

The approval of Institutional Review Board exempted the use of publicly available data.

## Results

### Study selection

**Figure 1:**
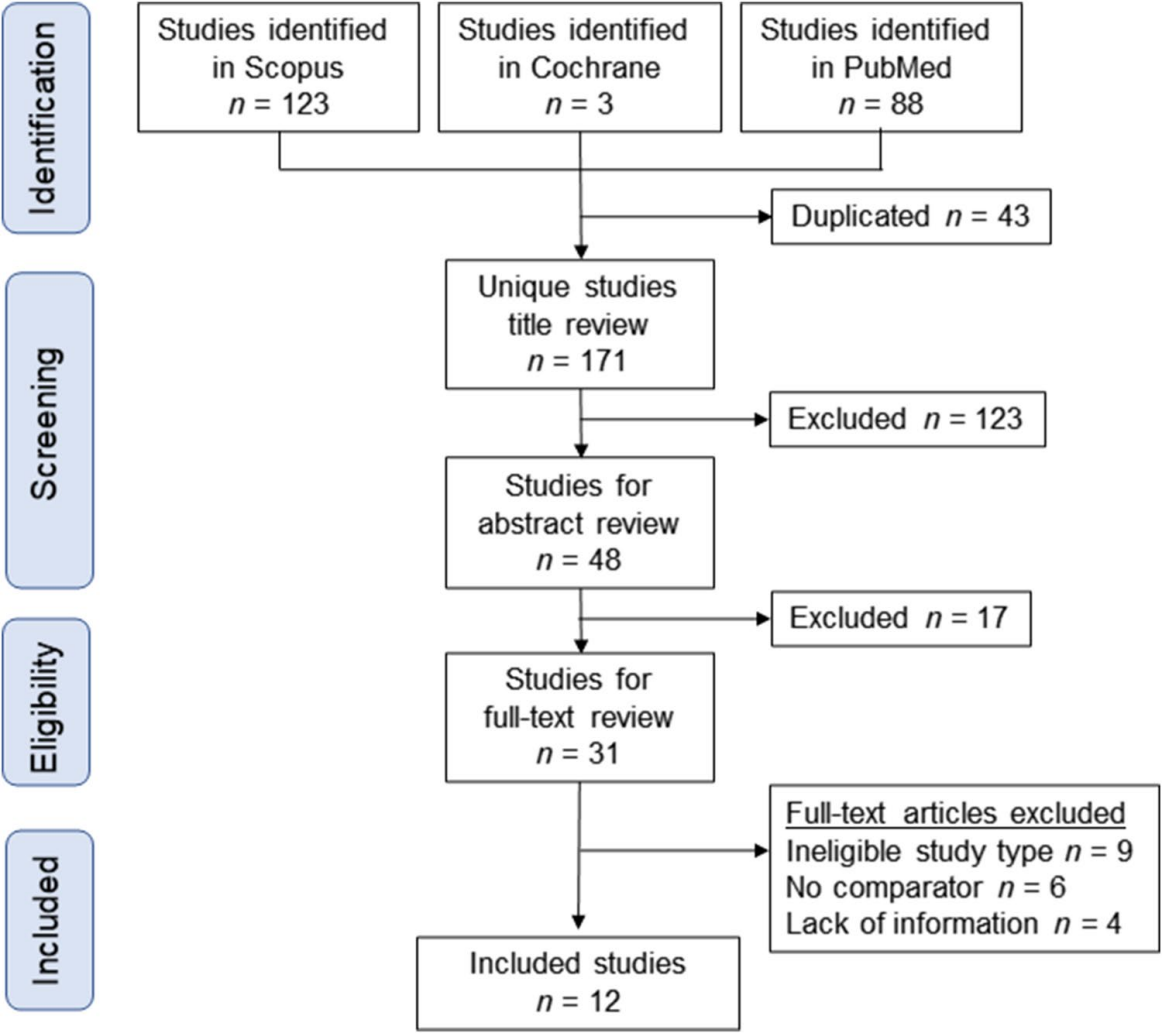
Study selection schema for the systematic review of the literature.

The study selection schema has been displayed in Fig. 1. A total of 214 studies were examined, and 12 studies, comprising of 190,139 IVF-ET pregnancies and 248,534 pregnancies with spontaneous conception met the inclusion criteria and were used for the descriptive analysis^10–12,15,32–39^.

#### Study characteristics

The meta-data of the evaluated studies are shown in Supplemental Table S2. One study was excluded due to the presence of overlapping cases^13^. The aims of the 12 studies (some studies were overlapping) were as follows: two studies examined the rate of antenatal diagnosis of PAS comparing between PAS after IVF-ET group and PAS with spontaneous conception group^15,39^, eight studies compared the effect of IVF-ET on PAS between IVF-ET group and spontaneous conception group^10–12,32,34,36–39^, three studies compared the prevalence of PAS between frozen ET and fresh ET group^11,33,36^, and one study compared the prevalence of PAS between frozen ET with hormone replacement cycle and frozen ET with normal ovulatory cycle^35^. The data on intracytoplasmic sperm injection were unavailable in most studies (*n* = 10).

No studies focused on the maternal outcomes of PAS after IVF-ET. Moreover, the severity (placenta accreta, increta, and percreta) of PAS was not compared between PAS after IVF-ET and PAS with spontaneous conception.

In the 12 included studies, the patients’ background was matched for maternal age in one study ^34^, and matched for maternal age and parity in the other^32^. Studies included in this review were published from 2011 to 2020. The majority of the studies were from Japan (58.3%)^10,15,32,33,35–37^, followed by United States (25.0%)^11,38,39^, China (8.3%)^34^, and Israel (8.3%)^12^.

#### Risk of bias of included studies

All studies were retrospective and of non-randomized comparative design^10–12,15,32–39^. No prospective studies were identified. The results of the risk of bias assessment for the comparative studies^10–12,15,32–39^ showed that there could be a moderate publication bias (moderate quality) in nine studies^10–12,32–35,38,39^ and severe publication bias (low quality) in three studies (Supplemental Table S3)^15,36,37^.

#### Definition of PAS

Among 12 studies, the definition of PAS was mentioned in six^11,12,15,36,38,39^. Of those, three studies defined PAS as a histopathological proven PAS^15,38,39^ and the remaining three defined PAS which was diagnosed by histopathological analysis or clinical diagnosis^11,12,36^.

### Meta-analysis

#### Diagnostic accuracy on PAS after IVF-ET

Two studies compared the antenatal diagnostic accuracy of PAS after IVF-ET (Table 2)^15,38^, one study determined the diagnostic accuracy of PAS after IVF-ET with placenta previa in MRI (28 cases of PAS)^15^, and another one investigated the rate of antenatal diagnosis of PAS without placenta previa in ultrasonography (112 cases of PAS)^39^. The rate of antenatal diagnosis of MRI for placenta previa patients with PAS after IVF-ET was significantly lower 2/9 (22.2%) than that for PAS with spontaneous conception 18/19 (94.7%, *P* < 0.01)^15^. Similar to the MRI study, the rate of antenatal diagnosis of ultrasonography for PAS after IVF-ET was significantly lower than that for PAS with spontaneous conception (< 4/31 [< 12.9%] *versus* 38/81 [46.9%], *P* < 0.01)^39^.

**Table 2:** Diagnostic accuracy of PAS after IVF-ET.

Author	Nagase^†^^15^			Modest^39^		
Year	2020			2020		
Previa	Yes			No		
No	Control	IVF	*P*-value	Control	IVF	*P*-value
Total	60	24		28,344	1418	
PAS	19 (31.6)	9 (37.5)	0.62	81 (0.3)	31 (2.2)	< 0.01
**Diagnosis**						
US	–	–	–	38/81 (46.9)	< 4/31 (< 12.9)^‡^	< 0.01
MRI	18/19 (94.7)	2/9 (22.2)	< 0.01	–	–	–

The numbers (percentages per column) are shown. ^†^This study included women who were evaluated for the presence of PAS by MRI. ^‡^As per the authors’ institutional requirement, cell size between 1 and 4 could not be reported.

*previa* placenta previa, *PAS* placenta accreta spectrum, *IVF* in vitro fertilization-embryo transfer, *US* ultrasonography, *MRI* magnetic resonance imaging.

These limited data suggested that the rate of antenatal diagnosis for PAS after IVF-ET may be lower than that of PAS with spontaneous conception both in women with and without placenta previa.

#### The risk of PAS between IVF-ET *versus* spontaneous conception

**Figure 2:**
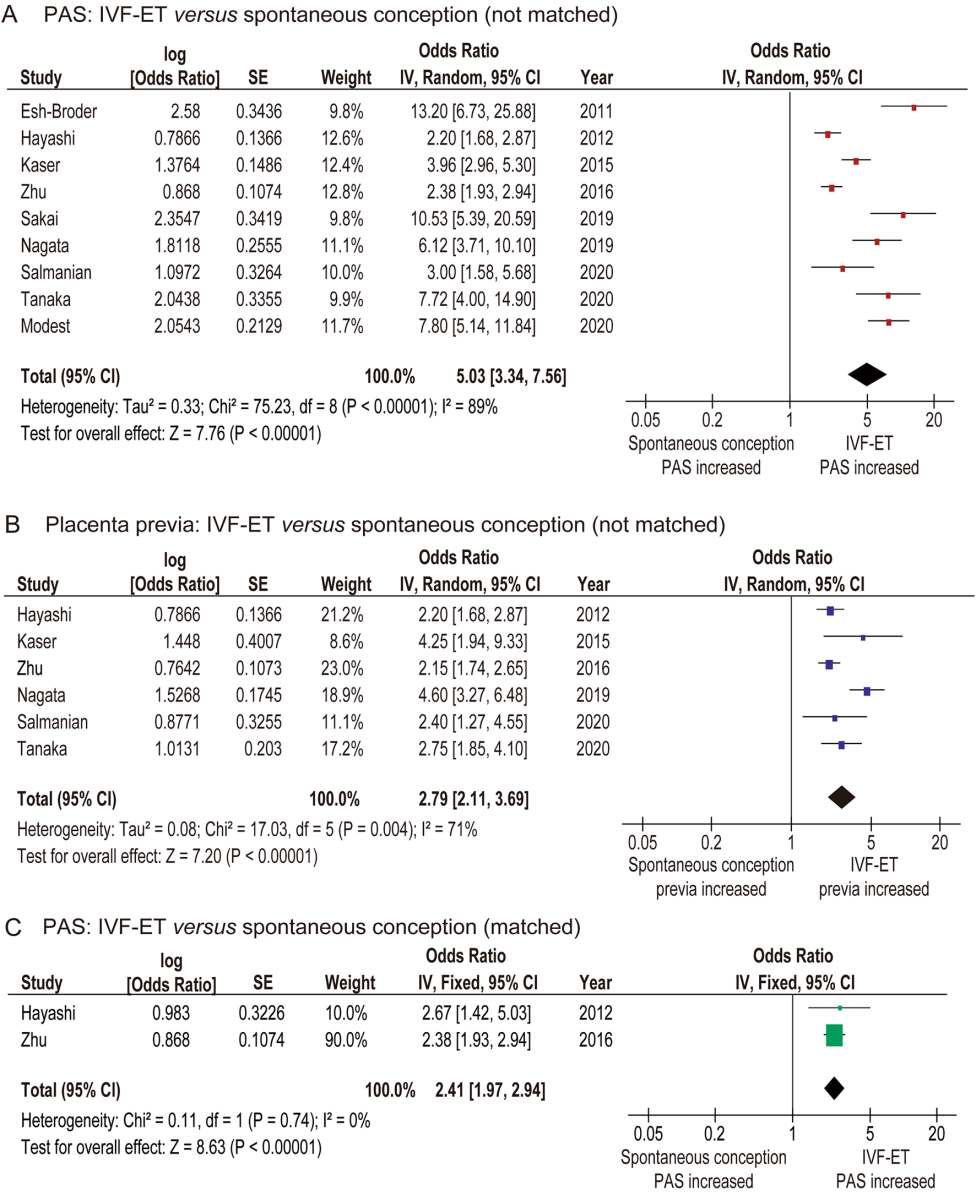
Results of the meta-analysis for the effect of IVF-ET on PAS. The pooled odds ratio for (**A**) PAS, (**B**) placenta previa, and (**C**) PAS (patient background matched) between IVF-ET patients *versus* spontaneous conception patients are shown. Since the numbers after the third decimal places for the lower or upper confidence interval were omitted in most studies, some calculated values of OR using Revman ver. 5.4.1™ may be different from the original values. Abbreviations: PAS, placenta accreta spectrum; OR, odds ratio; CI, confidence interval; and IVF-ET, in vitro fertilization-embryo transfer.

Nine studies (two of low and seven of moderate quality) examined the effect of IVF-ET on PAS (Table 3)^10–12,32,34,36–39^. In nine studies, there were 13,897 patients having IVF-ET pregnancies and 248,474 patients having pregnancies with spontaneous conception. Due to substantial heterogeneity, a random-effect analysis was performed. In the unadjusted pooled analysis (*n* = 9), IVF-ET was associated with an increased risk of PAS (OR: 5.03, 95%CI: 3.34–7.56, *P* < 0.01; heterogeneity: *P* < 0.01, *I*^*2*^ = 89%) (Fig. 2A). Egger’s test detected the presence of publication bias (*P* < 0.01). Of these nine studies, the effect of IVF-ET on placenta previa was examined in six. With regard to placenta previa, IVF-ET was associated with an increased risk of placenta previa (OR: 2.79, 95%CI: 2.11–3.69, *P* < 0.01; heterogeneity: *P* < 0.01, *I*^*2*^ = 71%) (Fig. 2B).

**Table 3:** Comparator analysis of PAS between women with IVF-ET and those with non-IVF-ET.

Author	Salmanian^38^	Modest^39^	Tanaka^37^	Sakai^36^	Nagata^10^	Zhu^34^	Kaser^11^	Hayashi^32^	Esh-Broder^12^
Year	2020	2020	2020	2019	2019	2016	2015	2012	2011
No	*n* = 37,461	*n* = 28,344	*n* = 6952	*n* = 735	*n* = 91,982	*n* = 7923	*n* = 54,947	*n* = 8834	*n* = 25,193
Control	36,890	26,926	6141	648	90,506	5282	53,376	4264	24,441
IVF	571	1418	811	87	1476	2641	1571	4570	752
Fresh	–	–	81	27^b^	–	–	1351	–	–
Frozen	–	–	730	60	–	–	220	–	–
ICSI	–	–	–	34/87 (39.1)	–	314/2641 (11.9)	–	–	–
**Previa**									
Control	271 (0.7)	–	96 (1.6)	–	489 (0.5)	179 (3.4)	–	–	–
IVF	10 (1.8)	–	34 (4.2)	–	36 (2.4)	185 (7.0)	OR: 4.25	OR: 2.20	–
**PAS**									
Matching	No	No	No	No	No	Age	No	^c^	No
Control	218 (0.6)	81 (0.3)	18 (0.3)	19 (2.9)	172 (0.2)	173 (3.3)	447 (0.8)		30 (0.1)
IVF	12 (2.1)	31 (2.2)	18 (2.6)	21 (24.1)	17 (1.15)	197 (7.5)	51 (3.2)	OR: 2.7	12 (1.6)
Definition	Path	Path	–	Path^a^, Clin	–	–	Path^a^, Clin	–	Path^a^, Clin
**Cycle**									
Fresh	–	–	–	2 (7.4)	–	–	34 (2.5)	–	–
Frozen	–	–	–	19 (31.7)	–	–	17 (7.7)	–	–

The numbers (percentages per column) are shown.

^a^Included cases without cesarean hysterectomy.

^b^Two cases underwent FET during the ovulation cycle.

^c^Controls matched for maternal age, parity, BMI, smoking, alcohol consumption, and pre-existing maternal diseases.

*Path* histopathological diagnosis, *clin* clinical diagnosis, –, not applicable; *definition* definition of placenta accreta spectrum, *matching* patient background matching, *previa* placenta previa, *OR* odds ratio, *PAS* placenta accreta spectrum, *IVF* in vitro fertilization-embryo transfer, *fresh* fresh embryo transfer, *frozen* frozen embryo transfer, *FET* frozem embryo transfer, *ICSI* Intracytoplasmic sperm injection.

In the patient background-matched comparator analysis of the prevalence of PAS between IVF-ET *versus* spontaneous conception, a fixed-effect model was used as no heterogeneity was observed between the studies. In the adjusted pooled analysis (*n* = 2), IVF-ET was associated with an increased risk of PAS (OR: 2.41, 95%CI: 1.97–2.94, *P* < 0.01; heterogeneity: *P* = 0.74, *I*^*2*^ = 0%) (Fig. 2C). The risk of publication bias could not be calculated due to the small number of studies.

#### The risk of PAS: spontaneous conception *versus* fresh ET *versus* frozen ET

**Figure 3:**
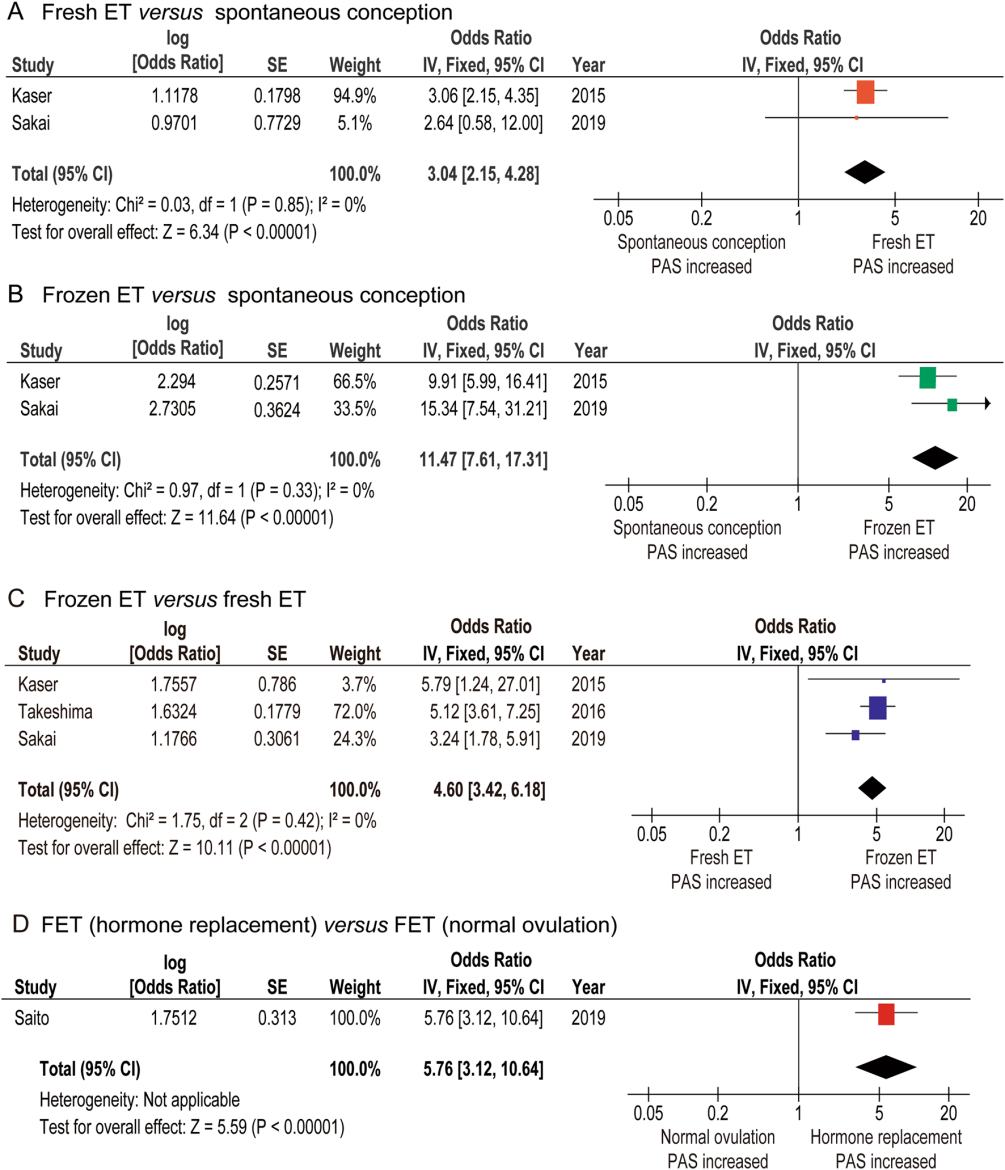
The effect of fresh ET, frozen ET, FET with normal ovulatory cycle, and FET with hormone replacement cycle on PAS. The pooled odds ratio for PAS **(A)** fresh ET *versus* spontaneous conception, **(B)** frozen ET *versus* spontaneous conception, **(C)** fresh ET *versus* frozen ET, and **(D)** frozen ET with hormone replacement cycle *versus* frozen ET with normal ovulatory cycle are shown. Since the numbers after the third decimal places for the lower or upper confidence interval are omitted in most studies, some calculated values of OR using Revman ver. 5.4.1™ may be different from the original values. *PAS* placenta accreta spectrum, *OR* odds ratio, *CI* confidence interval, *ET* embryo transfer, *FET* frozen embryo transfer.

To determine the risk of IVF-ET on PAS according to the type of IVF-ET (spontaneous conception *versus* fresh ET, spontaneous conception *versus* frozen ET, fresh ET *versus* frozen ET), a sensitivity analysis was performed. Two studies were identified to examine the risk of fresh ET or frozen ET on PAS compared to that on spontaneous conception^11,36^. Among the two studies, there were 1378 fresh ET patients, 280 frozen ET patients, and 54,024 spontaneous conception patients. A fixed-effect analysis was performed due to the absence of heterogeneity. In the comparator analysis between fresh ET *versus* spontaneous conception (*n* = 2), PAS patients were more likely to be observed in the fresh ET group compared to that of the spontaneous conception group (OR: 3.04, 95%CI: 2.15–4.28, *P* < 0.01; heterogeneity: *P* = 0.85, *I*^*2*^ = 0%) (Fig. 3A). Similarly, frozen ET was associated with an increased risk of PAS compared to that of spontaneous conception (OR: 11.47, 95%CI: 7.61–17.31, *P* < 0.01; heterogeneity: *P* = 0.33, *I*^*2*^ = 0%) (Fig. 3B). The risk of publication bias could not be calculated due to the small number of studies.

To determine whether fresh ET or frozen ET is a stronger risk factor of PAS, three studies were included in the analysis (one of low and two of moderate quality)^11,33,36^. Of those, there were 91,063 frozen ET patients and 51,833 fresh ET patients, respectively. Since no heterogeneity was observed, a fixed effect analysis was performed. In the pooled analysis (*n* = 3), frozen ET was associated with an increased risk of PAS (OR: 4.60, 95%CI: 3.42–6.18, *P* < 0.01; heterogeneity: *P* = 0.42, *I*^*2*^ = 0%) compared to that of fresh ET (Fig. 3C). The risk of publication bias could not be calculated due to the small number of studies. One study compared the rate of placenta previa between frozen ET *versus* fresh ET^33^. In this study, frozen ET was associated with a decreased rate of placenta previa compared to fresh ET (OR: 0.80, 95%CI: 0.70–0.89, *P* < 0.01).

#### The risk of PAS: frozen ET with hormone replacement cycle *versus* frozen ET with normal ovulatory cycle

One comparator study examined the prevalence of PAS between frozen ET with hormone replacement cycle *versus* frozen ET with normal ovulatory cycle groups (Table 4)^35^. This study did not include spontaneous conception patients and included only women with frozen ET. In this comparator analysis, frozen ET with hormone replacement cycle was significantly associated with the prevalence of PAS compared to that of frozen ET with normal ovulatory cycle (OR: 5.76, 95%CI 3.12–10.64, *P* < 0.01). The rate of placenta previa was similar between the two groups (OR: 0.80, 95%CI 0.59–1.08, *P* = 0.15).

**Table 4:** Comparator analysis of PAS between frozen ET *versus* fresh ET groups.

Author	Sakai^36^	Takeshima^33^	Kaser^11^	Saito^35^
Year	2019	2016	2015	2019
Area	JPN	JPN	USA	JPN
**No**				
Control	648	–	53,376	–
IVF	87	141,238	1571	34,980
Fresh	27 (31.0)	50,455 (35.7)	1351 (86.0)	–
Frozen	60 (69.0)	90,783 (64.3)	220 (14.0)	34,980
**Previa**				
Fresh	–	460 (0.9)	–	FET (normal): 66/10,755 (0.6)
Frozen	–	656 (0.7)	–	FET (hormone): 119/24,225 (0.5)
**PAS**				
Control	19 (2.9)	–	447 (0.8)	–
IVF	21 (24.1)	–	51 (3.2)	–
Definition	Path^a^, Clin	–	Path^a^, Clin	–
Fresh	2 (7.4)	35 (0.1)	34 (2.5)	0
Frozen	19 (31.7)	321 (0.4)	17 (7.7)	34,980
Normal	–	–	–	11/10,755 (0.1)
Hormone	–	–	–	142/24,225 (0.9)

Number (percentage per column) is shown. ^a^included cases without cesarean hysterectomy.

–, not applicable; *JPN*, Japan, *USA*, United States of America, *FET* frozen embryo transfer, *definition* definition of placenta accreta spectrum, *path* histopathological diagnosis, *clin* clinical diagnosis, *previa* placenta previa, *PAS* placenta accreta spectrum, *IVF* in vitro fertilization-embryo transfer, *fresh* fresh embryo transfer, *frozen* frozen embryo transfer, *hormone* hormone replacement cycle, *normal* normal ovulatory cycle.

## Discussion

### Main findings

The key findings of this study are the following: (i) the rate of antenatal diagnosis of PAS after IVF-ET may be lower than that of PAS with spontaneous conception, and (ii) IVF-ET was associated with an increased risk of PAS, especially in women who conceived with frozen ET with hormone replacement cycle.

### Comparison with existing literature

Several studies have reported that IVF-ET is associated with an increased risk of PAS. The first study that showed the relationship between IVF-ET and PAS was published in 2011^12^, but it did not determine the risk of PAS according to the type of IVF-ET^12^. Subsequently, Kaser et al*.* examined the risk of PAS in different groups (spontaneous conception [*n* = 53,376], fresh ET [*n* = 1,351], and frozen ET [*n* = 220])^11^. The authors found that women with an endometrial thickness of < 9 mm before ET had a greater risk of PAS than women with an endometrial thickness of ≥ 9 mm; thus, a thin endometrium may lead to PAS. In their analysis, the median endometrial thickness before ET was 8.4 mm in the frozen ET cycle and 10.6 mm in the fresh ET cycle. Therefore, frozen ET is considered a higher risk factor for PAS compared to that of fresh ET. Our systematic review found that Kaser et al*.*’s study is the only study that determined the association between endometrial thickness and PAS.

From the results of the meta-analysis, frozen ET with hormone replacement cycle appeared to be the most significant risk factor of PAS (Fig. 3A–D). Saito et al*.* compared the prevalence of PAS between women who conceived with frozen ET with hormone replacement cycle and those who conceived with frozen ET with normal ovulatory cycle^35^. They found that frozen ET with hormone replacement cycle is more likely to be complicated with PAS than frozen ET with normal ovulatory cycle. Although Saito et al*.*’s study did not examine the endometrial thickness, several studies have reported that endometrial thickness is lower in women who conceived with frozen ET with hormone replacement cycle than in those who conceived with frozen ET with normal ovulatory cycle or fresh ET^40–42^. Thus, our results are consistent with those of Kaser et al*.*’s study.

To discuss the relationship between IVF-ET and PAS, it is pertinent to mention that IVF-ET is associated with an increased rate of placenta previa, and placenta previa, in turn, is a high-risk factor for PAS (OR: 50–100)^6–8^. Therefore, it is possible that an increased risk of placenta previa contributes to an increased prevalence of PAS. A previous study that examined the relationship between endometrial thickness and placenta previa revealed that the risk of placenta previa is increased in women with an endometrial thickness of > 12 mm (adjusted OR: 3.74, 95%CI: 1.90–7.34) compared with women with an endometrial thickness of < 9 mm^42^. In contrast, Kaser et al.’s study has shown that an endometrial thickness of < 9 mm before ET had a greater risk of PAS^11^.

In our analysis, frozen ET was found to be associated with an increased risk of PAS compared to fresh ET, whereas the rate of placenta previa was not found to increase in the frozen ET group. Similarly, frozen ET with hormone replacement cycle is associated with an increased rate of PAS compared to frozen ET with normal ovulatory cycle, whereas the rate of placenta previa was similar between the two groups (Table 4). This study includes a comparator analysis to examine the association between IVF-ET and PAS. Since we did not focus on IVF-ET and placenta previa, our analysis missed a large study that compared the rate of placenta previa between women who conceived with fresh ET and frozen ET^43^.

A systematic review and meta-analysis that compared the risk of obstetrics complication showed no significant differences in the risk of placenta previa between the frozen ET and fresh ET groups (adjusted OR 0.70, 95%CI 0.46–1.08), which was consistent with our results^43^. These data support our findings suggesting that PAS can occur after IVF independently of the placental position.

We should note some limitations of this study in examining the effects of IVF-ET on the risk of PAS between women who conceived with IVF-ET and spontaneous conception. Advanced maternal age, increased parity, and prior cesarean delivery are the main risk factors for the development of placenta previa, and PAS in subsequent pregnancies has been reported in numerous large studies^44–48^. In general, women who require IVF to conceive are more often older and more likely to have a history of uterine surgery, including cesarean delivery or dilatation and curettage, compared with women who conceived with spontaneous conception^39,49–52^. We should note that these factors are the risk factors of placenta previa and PAS.

Since previous studies did not match the obstetric patient background or most studies did not perform a multivariate analysis with adjustments of various background factors in obstetrics patients, such as those mentioned above, our analysis cannot attribute IVF-ET as the risk factor of PAS while excluding the confounding factors.

We also should note that the quality of the diagnosis of PAS in this study may be low due to the following reasons: (i) all studies did not mention the severity or classification of PAS according to the previous literature^45,53^ and (ii) all studies did not report the detailed clinical findings at delivery and/or the histopathologic data. Therefore, there is a possibility that the present study has included women with less severe PAS (i.e., placenta adherent or creta) rather than women with the more severe type of PAS (i.e., placenta increta or percreta) ^53^. This is a strong limitation of this study, and further studies examining the effect of IVF-ET on the severity of PAS are warranted.

In summary, a lower endometrial thickness before ET may be associated with an increased risk of PAS; thus, confirmation of the endometrial thickness in women who have additional high-risk factors for PAS is recommended. Further studies investigating the relationship between endometrial thickness before ET and PAS are warranted.

The main cause of PAS, except for placenta previa, is damage to the endometrium-myometrial interface due to prior uterine surgeries, especially cesarean delivery^45,54^. These surgeries lead to abnormal decidualization in the area of the uterine scar, as well as myometrial invasion of the trophoblast and villi^47^. Previous studies have reported that an abnormally rich vascularity between the cesarean scar and placenta is observed in cases of PAS with uterine scars, even in early pregnancy. Unlike the typical cause of PAS with spontaneous conception, a thin endometrial thickness may be associated with PAS after IVF-ET^11^. It is speculated that abnormal vessels between the uterine scar and placenta are not newly developed in cases of PAS after IVF-ET without uterine scar; thus, the representative ultrasound or MRI findings are not observed due to the differences in the mechanisms of PAS. No studies have yet identified the specific findings of ultrasound or MRI in PAS after IVF-ET.

Although IVF-ET is associated with an increased risk of PAS, no study has yet compared the effects of IVF-ET on PAS in women with placenta previa. PAS complicated with placenta previa is one of the highest risk factors of massive postpartum hemorrhage^1,45,47^. Moreover, PAS after IVF-ET is associated with a lower rate of antenatal diagnosis. Therefore, if IVF-ET is associated with an increased risk of PAS in women with placenta previa, clinicians can be more aware of the presence of PAS in patients with placenta previa who have conceived with IVF-ET. On the other hand, since the technical ability for the antenatal diagnosis of PAS has been clearly shown in the literature^55–57^, we must appreciate the technical limitations of our diagnostic capabilities for PAS after IVF-ET.

Given the results of this study, it is important to anticipate that women who undergo IVF, especially frozen ET with hormone replacement cycle, are at increased risk for PAS which is difficult to identify antenatally, and some points as follows may be beneficial for the patients: (i) appropriate counseling prior to ET may be beneficial, as some women who have had successful pregnancies may not wish to assume this risk if known ahead of time, (ii) thorough and careful evaluations of the placenta by experienced sonologists and radiologists are key to identifying cases of PAS after IVF-ET, and (iii) preparations for the unidentified PAS after IVF-ET may help reduce hemorrhagic morbidity and improve prognosis, possibly because of multidisciplinary care, which includes cesarean hysterectomy, uterine artery embolization, transfusion preparation, and treatment by skilled physicians^18–22^.

### Strengths and limitations

The strengths of the study are that this is the first systematic review to focus on PAS after IVF-ET. Our study revealed that IVF-ET is associated with the prevalence of PAS, and this risk appeared to be highest in women who conceived with frozen ET with hormone replacement cycle. However, this study has some limitations. First, there was an unmeasured bias due to the retrospective studies we included in our systematic review. Potential sources of confounding variables in the study include the following: the varying definition of PAS across studies, the unmatched patient background, the lack of specific reasons for infertility, and the severity of PAS was not examined.

Second, the quality of the diagnosis of PAS appears to be low in most studies. Notably, all studies did not mention the severity of PAS and did not report the detailed clinical findings at delivery and/or the histopathologic data of PAS. Therefore, many of the studies included in the present study may have simple PAS rather than the severe one. This is a notable limitation of this study; hence, it should be considered while interpreting the results and the study's discussion for the patients having IVF-ET. Third, none of the studies matched the obstetric patient background to examine the effects of IVF-ET on the prevalence of PAS; thus, this study cannot isolate IVF-ET as the risk factor of PAS while excluding the confounding factors. This is an important limitation of this study.

Fourth, publication bias is a concern because the negative relationship between IVF-ET and PAS might not have been reported in the original papers. Egger’s test detected severe publication bias in the comparator analysis of the risk of PAS between IVF-ET *versus* spontaneous conception. This is another important limitation of this study. Fifth, only two studies examined the diagnostic accuracy of PAS after IVF-ET. To clearly show the accuracy of our findings, a more robust study should be conducted. Since a randomized controlled study is difficult due to the rarity of PAS after IVF-ET, a prospective study seems appropriate.

Lastly, the sample size was limited, particularly for the examination of the antenatal diagnosis for PAS after IVF-ET. Moreover, no prospective studies compared the prevalence of PAS among different groups (spontaneous conception *versus* fresh ET *versus* frozen ET with normal ovulatory cycle *versus* frozen ET with hormone replacement cycle).

### Conclusions

The antenatal diagnosis of PAS after IVF-ET was found to be significantly lower than that of PAS with spontaneous conception. Since IVF-ET is associated with an increased risk of PAS, we believe that clinicians can be more vigilant in ruling out “PAS after IVF-ET” in women with placenta previa who conceive with IVF-ET. To confirm the results of this study, further studies examining the diagnostic accuracy of PAS after IVF-ET are warranted. Future studies should also examine the risk of PAS among different types of IVF-ET to determine which type has the highest risk for PAS.

## Supplementary Information

Supplementary Information.

## Data Availability

All the studies used in this study are published in the literature.
