# Supplementary material for: Antenatal diagnosis of placenta accreta spectrum after in vitro fertilization-embryo transfer: a systematic review and meta-analysis
Source: Sci Rep. 2021 Apr 28;11:9205. doi: 10.1038/s41598-021-88551-7 (PMC8080594; doi:10.1038/s41598-021-88551-7)
Supplement: Supplementary file 1 — Supplementary Information. [file 41598_2021_88551_MOESM1_ESM.docx]

**Antenatal diagnosis of placenta accreta spectrum after *in vitro* fertilization-embryo transfer: A systematic review and meta-analysis**

Shinya Matsuzaki, MD, PhD^1,2,†^; Yoshikazu Nagase, MD^1,†^; Tsuyoshi Takiuchi, MD, PhD^1^; Aiko Kakigano, MD, PhD^3^; Kazuya Mimura, MD, PhD^1^; Misooja Lee, MD^1^; Satoko Matsuzaki, MD, PhD^1^; Yutaka Ueda, MD, PhD^1^; Takuji Tomimatsu, MD, PhD^1^; Masayuki Endo, MD, PhD^1,4^; Tadashi Kimura, MD, PhD^1^

^1^ Department of Obstetrics and Gynecology, Osaka University Graduate School of Medicine, Osaka, Japan

^2^ Department of Gynecology, Osaka International Cancer Institute, Osaka, Japan.

^3^ Department of Obstetrics and Gynecology, National Cerebral and Cardiovascular Center, Osaka, Japan

^4^ Department of Health Science, Osaka University Graduate School of Medicine, Osaka, Japan

^†^ Authors contributed equally

**Corresponding authors:**

Dr. Shinya Matsuzaki

Department of Obstetrics and Gynecology, Osaka University Graduate School of Medicine, 2-2 Yamadaoka, Suita, Osaka 565-0871, Japan

Telephone: +81-6-6879-3355; Fax: +81-6-6879-3359

E-mail: [zacky@gyne.med.osaka-u.ac.jp](mailto:zacky@gyne.med.osaka-u.ac.jp)

ORCID: <https://orcid.org/0000-0001-5725-9994>

**Word count:** Abstract 289 words, Main text 4,121 words

**Short title:** Placenta accreta spectrum after *in vitro* fertilization-embryo transfer

**Keywords:** placenta accreta spectrum, *in vitro* fertilization, diagnosis, systematic review

**Supplemental Table S1. The search strategy.**

PubMed

#1 “In Vitro Fertilization” [TIAB] 48,213

#2 fertilization in vitro [MeSH] 35,869

#3 Assisted Reproductive Techniques [MeSH] 69,788

#4 Embryo Transfer [MeSH] 16,146

#5 Intracytoplasmic Sperm Injection [MeSH] 6,647

#6 Cryopreserved [TIAB] 47,634

#7 "Oocyte donation" [TIAB] 3,174

#8 "fresh cycle" [TIAB] 185

#9 "frozen cycle" [TIAB] 34

#10 #1 OR #2 OR #3 OR #4 OR #5 OR #6 OR #7 OR #8 OR #9 119,226

#11 Placenta accreta [MeSH] 2,189

#12 "Morbidly adherent placenta" [TIAB] OR "Morbid adherent placenta" [TIAB] 250

#13 "Placenta Accreta Spectrum" [TIAB] 244

#14 "Placenta increta" [TIAB] OR "Placenta percreta" [TIAB] 860

#15 "adherence of placenta" [TIAB] OR "adherence of the placenta" [TIAB] OR "adherent placenta" [TIAB] 1,281

#16 #11 OR #12 OR #13 OR #14 OR #15 3,512

#17 #10 AND #16 88

Cochrane

#1 “In Vitro Fertilization”:ab,ti,kw 4,709

#2 MeSH descriptor: [fertilization in vitro] explode all trees 2,115

#3 MeSH descriptor: [Assisted Reproductive Techniques] explode all trees 3,266

#4 MeSH descriptor: [Embryo Transfer] explode all trees 1,112

#5 MeSH descriptor: [Intracytoplasmic Sperm Injection] explode all trees 570

#6 Cryopreserved:ab,ti,kw 897

#7 "Oocyte donation":ab,ti,kw 271

#8 "fresh cycle":ab,ti,kw 69

#9 "frozen cycle":ab,ti,kw 23

#10 #1 OR #2 OR #3 OR #4 OR #5 OR #6 OR #7 OR #8 OR #9 6,716

#11 MeSH descriptor: [Placenta accreta] explode all trees 26

#12 "Morbidly adherent placenta":ab,ti,kw OR "Morbid adherent placenta":ab,ti,kw 19

#13 "Placenta Accreta Spectrum":ab,ti,kw 6

#14 "Placenta increta":ab,ti,kw OR "Placenta percreta":ab,ti,kw 15

#15 "adherence of placenta":ab,ti,kw OR "adherence of the placenta":ab,ti,kw OR "adherent placenta":ab,ti,kw 28

#16 #11 OR #12 OR #13 OR #14 OR #15 60

#17 #10 AND #16 3

Scopus

#1 TITLE-ABS-KEY ("In Vitro Fertilization") 36,119

#2 TITLE-ABS-KEY ("fertilization in vitro") 47,055

#3 TITLE-ABS-KEY ("Assisted Reproductive Techniques" OR "Assisted Reproductive Technology") 13,273

#4 TITLE-ABS-KEY ("Embryo Transfer") 30,120

#5 TITLE-ABS-KEY ("Intracytoplasmic Sperm Injection") 17,021

#6 TITLE-ABS-KEY ("Cryopreserved ") 17,064

#7 TITLE-ABS-KEY ("oocyte donation") 4,022

#8 TITLE-ABS-KEY ("fresh cycle") 428

#9 TITLE-ABS-KEY ("frozen cycle") 168

#10 #1 OR #2 OR #3 OR #4 OR #5 OR #6 OR #7 OR #8 OR #9 96,894

#11 TITLE-ABS-KEY ("placenta accreta") 4,062

#12 TITLE-ABS-KEY ("Morbidly adherent placenta") OR TITLE-ABS-KEY ("Morbid adherent placenta") 324

#13 TITLE-ABS-KEY ("placenta accreta spectrum") 281

#14 TITLE-ABS-KEY ("Placenta increta" OR "placenta percreta”) 1,128

#15 TITLE-ABS-KEY ("adherence of placenta" OR "adherence of the placenta" OR "adherent placenta") 528

#16 #11 OR #12 OR #13 OR #14 OR #15 4,438

#17 #10 AND #16 123

**Supplemental Table S2. Meta-table of the included studies.**

| Author | Nagase | Salmanian | Modest | Tanaka | Sakai | Saito | Nagata | Zhu | Takeshima | Kaser | Hayashi | Esh-Broder |
| --- | --- | --- | --- | --- | --- | --- | --- | --- | --- | --- | --- | --- |
| Reference | [15] | [37] | [38] | [36] | [35] | [34] | [10] | [33] | [32] | [11] | [31] | [12] |
| Year | 2020^¶^ | 2020 | 2020 | 2020 | 2019 | 2019 | 2019 | 2016 | 2016 | 2015 | 2012 | 2011 |
| Area | JPN | USA | USA | JPN | JPN | JPN | JPN | CHN | JPN | USA | JPN | ISR |
| No. | *n*=84 | *n*=37461 | *n*=28344 | *n*=6952 | *n*=735 | *n*=34980 | *n*=91982 | *n*=7923 | *n*=141238 | *n*=54947 | *n*=8834 | *n*=25193 |
| Matching | No | No | No | No | No | No | No | Yes (1:2) | No | No | Yes | No |
| Control | 60 | 36890^§^ | 26926 | 6141 | 648 | -- | 90506 | 5282 | -- | 53376 | 4264 | 24441 |
| IVF | 24 | 571 | 1418 | 811 | 87 | 34980 | 1476 | 2641 | 141238 | 1571 | 4570 | 752 |
| Fresh | -- | -- | -- | 81 | 27** | -- | -- | -- | 50455 | 1351 | -- | -- |
| Frozen | -- | -- | -- | 730 | 60 | 34980 | -- | -- | 90783 | 220 | -- | -- |
| Previa |  |  |  |  |  |  |  |  |  |  |  |  |
| Control | 19 (31.7) | 271 (0.7) | -- | 96 (1.6) | -- | 66 (0.6)^#^ | 489 (0.5) | 179 (3.4) | 460 (0.9)^#^ | OR: 4.25 | OR: 2.20 | -- |
| IVF | 9 (37.5) | 10 (1.8) | -- | 34 (4.2) | -- | 119 (0.5)^$^ | 36 (2.4) | 185 (7.0) | 656 (0.7)^$^ |  |  | -- |
| PAS |  |  |  |  |  |  |  |  |  |  |  |  |
| Control | 19 (31.7) | 218 (0.6) | 81 (0.3) | 18 (0.3) | 19 (2.9) | -- | 172 (0.2) | 173 (3.3) | -- | 447 (0.8) | OR: 2.67 | 30 (0.1) |
| IVF | 9 (37.5) | 12 (2.1) | 31 (2.2) | 18 (2.6) | 21 (24.1) | -- | 17 (1.2) | 197 (7.5) | -- | 51 (3.2) |  | 12 (1.6) |
| Definition | Path | Path | Path | -- | Path*, Clin | -- | -- | -- | -- | Path*, Clin | -- | Path*, Clin |
| PAS (type of IVF) | |  |  |  |  |  |  |  |  |  |  |  |
| Fresh | -- | -- | -- | -- | 2 (7.4) | 0 | -- | -- | 35 (0.1) | 34 (2.5) | -- | -- |
| Frozen | -- | -- | -- | -- |  |  | -- | -- |  |  | -- | -- |
| Total | -- | -- | -- | -- | 19 (31.7) | 34980 | -- | -- | 321 (0.4) | 17 (7.7) | -- | -- |
| Normal | -- | -- | -- | -- | -- | 11/10755 (0.1) | -- | -- | -- | -- | -- | -- |
| Hormone | -- | -- | -- | -- | -- | 142/24225 (0.9) | -- | -- | -- | -- | -- | -- |
| Diagnosis |  |  |  |  |  |  |  |  |  |  |  |  |
| US | -- | -- | <4/31 vs 38/81 | -- | -- | -- | -- | -- | -- | -- | -- | -- |
| MRI | 2/9 vs 18/19 | -- | -- | -- | -- | -- | -- | -- | -- | -- | -- | -- |

^*^Included cases without cesarean hysterectomy. ^**^Two cases underwent FET during the ovulation cycle. ^#^Fresh cycle. ^$^Frozen cycle. ^¶^Women who underwent MRI for antenatal evaluation of the placenta accreta spectrum during the study period were included. ^§^Calculated by the data of patient background. Some values listed above might be slightly different from the original values due to estimation by the authors. Abbreviations: --, not applicable; OR, odds ratio; Definition, definition of placenta accreta spectrum; Path, histopathological diagnosis; Clin, clinical diagnosis; previa, placenta previa; PAS, placenta accreta spectrum; IVF, *in vitro* fertilization-embryo transfer; FET, frozen embryo transfer; US, ultrasonography; MRI, magnetic resonance imaging; Hormone, frozen embryo transfer with hormone replacement cycle; Normal, frozen embryo transfer with normal ovulatory cycle; JPN, Japan; USA, United States of America; CHN, China; ISR, Israel.

**Supplemental Table S3. Risk of bias assessment for the comparator study.**

| Authors | Confounding | Selection | Classification of intervention | Deviations from interventions | Missing data | Measurement of outcomes | Reported results | Overall bias |
| --- | --- | --- | --- | --- | --- | --- | --- | --- |
| Nagase | ● | ● | ● | ● | ● | ● | ● | ● |
| Salmanian | ● | ● | ● | ● | ● | ● | ● | ● |
| Modest | ● | ● | ● | ● | ● | ● | ● | ● |
| Tanaka | ● | ● | ● | ● | ● | ● | ● | ● |
| Sakai | ● | ● | ● | ● | ● | ● | ● | ● |
| Saito | ● | ● | ● | ● | ● | ● | ● | ● |
| Nagata | ● | ● | ● | ● | ● | ● | ● | ● |
| Zhu | ● | ● | ● | ● | ● | ● | ● | ● |
| Takeshima | ● | ● | ● | ● | ● | ● | ● | ● |
| Kaser | ● | ● | ● | ● | ● | ● | ● | ● |
| Hayashi | ● | ● | ● | ● | ● | ● | ● | ● |
| Esh-Broder | ● | ● | ● | ● | ● | ● | ● | ● |

Risk of bias assessment was performed using the Risk Of Bias In Non-randomized Studies–of Interventions tool (ROBINS-I) ^28-30^.

● Low risk of bias (the study is comparable to a well-performed randomized trial with regard to this domain)

● Moderate risk of bias (the study is sound for a non-randomized study with regard to this domain but cannot be considered comparable to a well-performed randomized trial)

● Serious risk of bias (the study has some important problems in this domain)

● Critical risk of bias (the study is too problematic in this domain to provide any useful evidence on the effects of intervention)

● No information on which to base a judgement about risk of bias for this domain.
